# Commentary: On the Importance of the Speed-Ability Trade-Off When Dealing With Not Reached Items

**DOI:** 10.3389/fpsyg.2018.01988

**Published:** 2018-10-30

**Authors:** Steffi Pohl, Matthias von Davier

**Affiliations:** ^1^Department of Education and Psychology, Freie Universität Berlin, Berlin, Germany; ^2^National Board of Medical Examiners, Philadelphia, PA, United States

**Keywords:** missing values, response time, not reached items, speed-ability trade-off, time limit, speed-accuracy

In their 2018 article, (T&B) discuss how to deal with not reached items due to low working speed in ability tests (Tijmstra and Bolsinova, 2018). An important contribution of the paper is focusing on the question of how to define the targeted ability measure. In this note, we aim to add further aspects to this discussion and to propose alternative approaches.

## Challenges in estimating optimal ability

### Ignoring the dimensional structure

To show effects of too low working speed, T&B (p. 6) consider a model combining effective working speed and optimal ability

(1) $$\begin{array}{r} P_{i}\left(X=1|\theta^{*},\tau \right)=\frac{1}{1+\exp\left(-\alpha_{i}\left[\theta_{p}^{*}+\gamma_{p}\left(\tau^{*}-\tau_{p}\right)-\beta_{i}\right]\right)}. \end{array}$$

T&B assume two respondent groups: Compliers with $\tau_{p}=\tau^{*}$ and non-compliers with lower than optimal working speed, i.e., $\tau^{*}<\tau_{p}$ which implies $\gamma_{p}\left(\tau^{*}-\tau_{p}\right)<0$ if γ_*p*_ > 0. We refer to this group as slow non-compliers (slowNCs).

For compliers (with $\tau^{*}=\tau_{p}$), the model in (1) reduces to a one-dimensional IRT model since $\gamma_{p}\left(\tau^{*}-\tau_{p}\right)=0$. For non-compliers, defining α_1*i*_ = α_*i*_, α_2*ip*_ = −γ_*p*_α_*i*_ and $\beta_{ip}^{*}=\alpha_{i}\left(\gamma_{p}\tau^{*}-\beta_{i}\right)$, a person-specific two-dimensional IRT model depending on the speed-ability trade-off (SAbT) parameter γ_*p*_ results, i.e.,

(2) $$\begin{array}{r} P_{i}\left(X=1|\theta^{*},\tau \right)=\frac{1}{1+\exp\left(-\left[{\alpha_{1i}\theta }^{*}+\alpha_{2ip}\tau +\beta_{ip}^{*}\right]\right)}. \end{array}$$

Apart from specific experimental settings, which are rarely feasible to implement in large-scale assessments, in practice this model cannot be estimated, so T&B resort to fixing γ_*p*_ to a constant for their simulations. This specifies a regular two-dimensional IRT for simulation, and using a unidimensional model for analysis will of course result in biased ability estimates, which can be quantified as follows

(3) $$\begin{array}{r} E\left(\hat{\theta_{p}}\right)=\theta_{p}^{{}^{*}}+\gamma_{p}\left(\tau^{*}-\tau_{p}\right). \end{array}$$

Only compliers with $\tau^{*}=\tau_{p}$ or respondents with γ_*p*_ = 0 would obtain unbiased person parameter estimates from a unidimensional model. Thus, bias is not a result of how missing responses are treated, but due to ignoring the dimensional structure.

### Respondents faster than optimal

T&B only consider non-compliance as lower speed than optimal. However, most of the non-complying respondents show higher speed than optimal. Even respondents who manage responding to all items within the time limit will not have speed $\tau_{p}=\tau^{*}$, but $\tau_{p}>\tau^{*}$. This was noted by Kuhn and Ranger (2015) and shown in our own empirical data analyses (up to 70% of respondents without missing values finish the test some time before the time limit; Pohl, 2018; Pohl et al., under review; Ulitzsch et al., under review). Thus, a third group is needed in this discussion, which we will call faster non-compliers (fastNCs). Note that fastNCs—who will likely reach all items—will also receive biased estimates according to Equation (3). Hence, the issue of estimating optimal ability cannot solely be solved by focusing on the treatment of missing values.

## Evaluation of missing data approaches

### Assumption on the missing data process

When evaluating the performance of approaches for estimating optimal ability, one must consider a more realistic missing data mechanism including that (a) there is fastNC and (b) not reached items also occur due to quitting. In fact, in low stakes assessments quitting seems to be the main reason for not reached items (up to 90% of not reached items are due to quitting, see Pohl, 2018; Pohl et al., under review; Ulitzsch et al., under review). This will alter the results.

### Performance of the missing data treatments

T&B conclude that incorrect scoring shows the best results compared to other approaches. First, T&B's result seems somewhat surprising since the finding on the performance of incorrect scoring stands in stark contrast to other published research on this approach (Lord, 1974; De Ayala et al., 2001; Rose et al., 2010; Pohl et al., 2014) which show that incorrect scoring results in highly biased parameter estimates whenever missing values do not only occur on otherwise incorrect responses. Second, note that scoring missing values as incorrect results in a different definition of the target ability for different subgroups. For slowNCs with missing values, scoring these as incorrect results in an overcorrection for speed while aiming at estimating optimal ability. For compliers and fastNCs no corrections for speed are made, as there are no missing data, but instead effective ability is estimated.

## Discussion of proposed solutions

We appreciate the solutions proposed by T&B and want to add further aspects for consideration:

Non-speeded power tests rely on respondents (a) being aware of their own SAbT function and (b) being highly motivated to optimize performance. The first assumption is unlikely to hold in many applications. The second assumption may hold in high stakes assessments, while in low stakes assessments, for which the missing data approaches have been suggested, empirical data (e.g., Cosgrove and Cartwright, 2014; Pohl et al., under review); suggest otherwise. Also note that this solution requires moving from measuring optimal ability for a given time limit and instead opt for measuring effective ability given the chosen speed.

Item-level time limits help respondents to manage time and reduce variability in chosen speed. However, note that this solution (a) cannot resolve the issue of differences in speed across respondents as there will still be fastNCs and (b) induces other problems, as for example increased item omit rates or rapid guessing.

## An alternative solution

One may conjecture that effective speed and effective ability more closely mirror real life behavior, which is typically the goal in large scale assessments (OECD, 2017). These may even be better predictors for later outcomes than optimal ability: In everyday situations there is no information on optimal speed but persons typically chose their speed given external time limits.

Pohl et al., under review and Ulitzsch et al., under review suggest describing performance of respondents by the *profile of all dimensions of performance*: effective ability, effective speed, and test endurance (as a measure of quitting behavior) and to use these dimensions for evaluating and comparing performance. This allows developing a richer description of differences in performance and to disentangle the different aspects involved. This also allows explaining differences in performance (e.g., Sachse et al., in preparation). If stakeholders are interested in only one score per domain, as for example for country rankings, we suggest using a constructive approach and decide either empirically (through prediction of key outcomes) or by means of a validity argument how to combine ability, speed, and test endurance by developing a composite score that reflects the combination one wants to focus on. One advantage of such an approach would be that this composite is the same for all respondents (not just for those with missing values). Note that this solution also works for omitted responses; these just need a slightly different modeling approach (Ulitzsch et al., 2018; Ulitzsch et al., under review).

## Author contributions

SP wrote the first draft of the manuscript including the general outline of argumentation. MvD discussed these with SP and added further ideas. SP and MvD both revised the manuscript.

### Conflict of interest statement

The authors declare that the research was conducted in the absence of any commercial or financial relationships that could be construed as a potential conflict of interest.
